# Mutations in *NDUFS1* Cause Metabolic Reprogramming and Disruption of the Electron Transfer

**DOI:** 10.3390/cells8101149

**Published:** 2019-09-25

**Authors:** Yang Ni, Muhammad A. Hagras, Vassiliki Konstantopoulou, Johannes A. Mayr, Alexei A. Stuchebrukhov, David Meierhofer

**Affiliations:** 1 Mass Spectrometry Facility, Max Planck Institute for Molecular Genetics, 14195 Berlin, Germany; yang.ni@kuleuven.vib.be; 2 Department of Biology, Chemistry and Pharmacy, Freie Universität Berlin, 14195 Berlin, Germany; 3 Present address: Laboratory of Angiogenesis and Vascular Metabolism, VIB-KU Leuven Center for Cancer Biology, 3000 Leuven, Belgium; 4 Department of Chemistry, University of California Davis, Davis, CA 95616, USA; mahagras@ucdavis.edu (M.A.H.); aastuchebrukhov@ucdavis.edu (A.A.S.); 5 Present address: Department of Chemical Engineering, Massachusetts Institute of Technology, Cambridge, MA 02142, USA; 6 Department of Pediatrics and Adolescent Medicine, Medical University of Vienna, 1090 Vienna, Austria; vassiliki.konstantopoulou@meduniwien.ac.at; 7 Department of Pediatrics, Paracelsus Medical University Salzburg, 5020 Salzburg, Austria; H.Mayr@salk.at

**Keywords:** complex I (CI) deficiency, metabolome and proteome profiling, reactive oxygen species (ROS), respirasome assembly, electron tunneling (ET)

## Abstract

Complex I (CI) is the first enzyme of the mitochondrial respiratory chain and couples the electron transfer with proton pumping. Mutations in genes encoding CI subunits can frequently cause inborn metabolic errors. We applied proteome and metabolome profiling of patient-derived cells harboring pathogenic mutations in two distinct CI genes to elucidate underlying pathomechanisms on the molecular level. Our results indicated that the electron transfer within CI was interrupted in both patients by different mechanisms. We showed that the biallelic mutations in *NDUFS1* led to a decreased stability of the entire N-module of CI and disrupted the electron transfer between two iron–sulfur clusters. Strikingly interesting and in contrast to the proteome, metabolome profiling illustrated that the pattern of dysregulated metabolites was almost identical in both patients, such as the inhibitory feedback on the TCA cycle and altered glutathione levels, indicative for reactive oxygen species (ROS) stress. Our findings deciphered pathological mechanisms of CI deficiency to better understand inborn metabolic errors.

## 1. Introduction

Complex I (CI, NADH:ubiquinone oxidoreductase) is the first and largest enzyme of the mitochondrial respiratory chain in humans. It catalyzes the transfer of electrons from NADH to coenzyme Q10, which is coupled to the translocation of protons from the mitochondrial matrix into the intermembrane space. Recently, the structures of the entire CI in *Yarrowia lipolytica*, *Ovis aries*, and *Bos taurus* were reported at a resolution of 3.6 to 4.2 Å, describing in detail the central subunits that execute this bioenergetic function [1,2,3]. Mammalian CI consists of 45 subunits, seven of which are encoded by the genes localized in mitochondrial DNA [4,5,6]. Therefore, CI deficiency can originate from both mitochondrial or nuclear DNA mutations, which leads to its heterogeneous features [7,8].

Since the discovery of pathogenic mitochondrial DNA (mtDNA) point mutations [9,10] and deletions [11] in the year 1988, more than 309 gene defects have been reported to date, and this number continues to grow [12]. Mitochondrial diseases can be grouped into (i) disorders of oxidative phosphorylation (OXPHOS) subunits and their assembly factors; (ii) defects of mitochondrial DNA, RNA, and protein synthesis; (iii) defects in the substrate-generating upstream reactions of OXPHOS; (iv) defects in relevant cofactors; and (v) defects in mitochondrial homeostasis [13]. Mitochondrial diseases occur at an estimated prevalence of 1 in 5000 live births, and are collectively the most common inborn error of metabolism [14,15]. CI deficiency is the most frequent mitochondrial disorder among inborn errors of metabolism, and is characterized by clinical and genetic heterogeneity [16] including Leber’s hereditary optic neuropathy (LHON) [10], mitochondrial encephalomyopathy, lactic acidosis, stroke-like episodes (MELAS) [17], and Leigh syndrome (LS) [18]. In addition, the level of heteroplasmy of mtDNA mutations can vary and is dynamic between cells in the same organism or tissue, and the proportion of mutant mtDNA molecules determines both the penetrance and severity of expression of disease [19]. Recently, Idebenone was designated as the first orphan drug to treat LHON by the European Medicines Agency (EMA product number: EMEA/H/C/3834). Idebenone functions as a mitochondrial electron carrier and bypasses CI to directly transfer electrons to mitochondrial complex III (CIII) [20].

In this study, we applied an integrative proteome and metabolome profiling approach to investigate the molecular and cellular consequences of pathogenic mutations in two core subunits of mitochondrial CI. The first nuclear gene, *NDUFS1*, encodes the NADH-ubiquinone oxidoreductase 75 kDa subunit, the largest subunit of CI that accommodates three iron–sulfur clusters in the N-module, which binds and oxidizes NADH [21,22]. The second gene, *MT-ND5*, is located in the mtDNA and encodes NADH-ubiquinone oxidoreductase chain 5, which represents one of the core subunits in the P-module, wherein the proton translocation takes place. It is located at the distal end of the CI transmembrane arm and facilitates proton translocation [23,24].

The first patient was a girl, who carried a mutation in the mitochondrial gene *MT-ND5* (m.12706T>C). This missense mutation caused a single amino acid substitution of p.Phe124Leu. The second patient was a boy. He carried point mutations in *NDUFS1* (c.683T>C and 755A>G, compound heterozygous), which caused amino acid substitutions of p.Val228Ala and p.Asp252Gly. Identical mutations in both genes have been previously reported to cause a pathogenic phenotype [25,26,27,28,29,30]; however, the molecular and cellular consequences of these mutations were largely unknown. Here, we explored and compared the proteome and metabolome profiles of patients and control skin fibroblasts to elucidate (i) if the global and OXPHOS-specific protein and metabolite abundances were altered, (ii) if the assembly of CI and the formation of the mitochondrial respirasome was influenced, (iii) if enzymatic activities of OXPHOS were regulated, (iv) if reactive oxygen species (ROS) production was changed in these distinct CI mutations versus unaffected controls, and (v) whether the electron tunneling rate in NDUFS1 was impaired because of the mutation between iron–sulfur clusters N4 and N5.

## 2. Materials and Methods

### 2.1. Ethics Statement

The study protocol conformed to the guidelines of the Declaration of Helsinki. Studies with primary human cell lines were approved by the local ethics committee “Ethikkommission Land Salzburg” and written informed consent was provided by the patients’ guardians for skin biopsies.

### 2.2. Mutations in Patients

The first patient carried a missense mutation in the mitochondrial DNA (gene *MT-ND5*, m.12706T>C), which lead to an amino acid substitution (p.Phe124Leu) in the ND5 subunit of complex I. Sanger sequencing revealed a 70% mutation load in cultivated skin fibroblasts. This mutation has been reported in patients with Leigh syndrome [25,26,27]. The second patient carried two distinct point mutations (c.[683T>C];[755A>G]) in the nuclear gene *NDUFS1* (NM_005006.7), which caused amino acid substitutions (p.[Val228Ala];[Asp252Gly]) in the NDUFS1 subunit of complex I. This patient was compound heterozygous for these two missense mutations. 

### 2.3. Patients

Patient *MT-ND5*: During pregnancy aortic stenosis was diagnosed by sonography. The mother suffered from epilepsy and was treated with Levetiracetam during pregnancy. On the first day of life, the girl presented with right ventricular hypertrophic cardiomyopathy. Sonography of the brain showed partial agenesis of the corpus callosum. At the age of 9 months and during an upper airway infection, the girl was admitted to intensive care because of apnea and insufficient spontaneous breathing. In addition, a brain magnetic resonance imaging showed symmetric signal alterations in the basal ganglia and the brain stem. At the age of 9½ months, the girl died from respiratory failure. Investigation of an unfrozen muscle biopsy revealed a decrease in CI: 20 nmol/min/mg protein (normal 28–76 nmol/min/mg protein, Table 1). Lactate was elevated in blood between 41–50 mg/dL (normal 6–22 mg/dL). Urine organic acid analysis revealed elevated lactate (262 mmol/mol creatinine), 3-hydroxybutyrate, and acetoacetate.

Patient *NDUFS1*: At the age of 7 months, this boy presented with muscular hypotonia. He lost skills such as head control and rolling over. Furthermore, he failed to thrive and lost body weight. A muscle biopsy was performed at the age of 9 months and showed decreased activity of respiratory chain CI: 20 nmol/min/mg protein (normal 28–76 nmol/min/mg protein, Table 1).

### 2.4. Cell Culture

Human-derived primary skin fibroblast cells (patients and controls) were obtained from the Department of Pediatrics, Salzburger Landeskliniken, Salzburg, Austria. Fibroblasts were obtained by a superficial punch skin biopsy, collected from the patient under local anesthesia. Two individuals, from whom the skin fibroblasts were taken as controls, had no genetic defects and were hospitalized for sepsis. In order to meet the requirement of individual experiments, cells were grown in slightly different media. For proteome and metabolome profiling, as well as blue native-polyacrylamide gel electrophoresis (BN-PAGE), cell lines were cultivated in high glucose Dulbecco’s modified eagle medium (DMEM, Thermo Scientific, Waltham, MA, USA, # 31966) containing 4.5 g/L glucose, 1 mM pyruvate, and GlutaMAX, and supplemented with 10% fetal bovine serum (FBS, Merck, Darmstadt, Germany, # F7524), 1% penicillin-streptomycin-neomycin (PSN) antibiotic mixture (Thermo, # 15640055) at 37 °C in a normoxia incubator with a humidified atmosphere of 5% CO_2_. Cells were grown to 90% confluency in one T75 or one T300 polystyrene flask (TPP, Trasadingen, Switzerland) in biological triplicates for proteome and metabolome experiments, respectively. For the live-cell respiration assay with the Seahorse XFe96 Analyzer (Agilent, Santa Clara, CA, USA), fibroblasts were cultured in basic DMEM (Thermo, # A14430), supplemented with 1 mM sodium pyruvate (Merck, # P2256), 2 mM L-glutamine (Merck, # G3126), 1% PSN antibiotic mixture (Thermo, # 15640055), 10% dialyzed FBS (Silantes, # 281000900), and 25 mM glucose (Merck, # G7021) in three biological replicates in T150 flasks.

### 2.5. Metabolite Extraction and Profiling by Targeted LC-MS

Metabolite extraction was done as reported previously with a minor modification for cell culture samples [32]. In brief, T300 flasks with fibroblasts between the passages of 11–15 at 90% confluence were harvested in triplicates for each experiment. Twenty-four hours before harvest, the cell culture was replenished with fresh medium. In order to keep the original metabolic state of the cell and minimize metabolite degradation, cells were harvested within 2 minutes. Culture medium was aspirated and the cells were rinsed quickly twice with ice-chilled 1× phosphate-buffered saline, pH 7.4. Then, 1 mL water was added into the flask, which was immediately shock-frozen in liquid nitrogen. The flask was kept on ice, and cell lysates were collected with a cell scraper (TPP) and transferred into a 15 mL tube for three thaw-and-freeze cycles to extract the metabolites. Metabolites were extracted with methyl tert-butyl ether (MTBE), methanol, and water [32]. The remaining protein pellet was used in the bicinchoninic acid (BCA) protein assay for normalization among samples. Extracts were aliquoted equally into three tubes for later reconstitution in water, acetonitrile, and 50% methanol in acetonitrile, respectively. Additionally, an internal standard mixture, containing chloramphenicol and C^13^-labeled L-glutamine, L-arginine, L-proline, L-valine, and uracil was added to each sample (10 µM final concentration). A SpeedVac was used to dry the aliquots. Dry residuals were dissolved in three different solvents (1) 100 µL 50% acetonitrile in MeOH with 0.1% formic acid, (2) 100 µL MeOH with 0.1% formic acid for analysis by hydrophilic interaction liquid chromatography (HILIC) column, or (3) 100 µL water with 0.1% formic acid for C18 column mode. The supernatants were transferred to micro-volume inserts. Then, 20 µL per run was injected for subsequent LC-MS analysis.

Over 400 metabolites were selected to cover most of the important metabolic pathways in mammals. Metabolites are very diverse in their chemical properties. Therefore, two different LC columns have been used for metabolite separation: a Reprosil-PUR C18-AQ (1.9 µm, 120 Å, 150 × 2 mm ID; Dr. Maisch, Ammerbuch, Germany) column, and a zicHILIC (3.5 µm, 100 Å, 150 × 2.1 mm ID; Merck). The settings of the LC-MS instrument, 1290 series ultra high pressure liquid chromatography (UHPLC) (Agilent) online coupled to a QTRAP 6500 (Sciex, Foster City, CA) were reported previously [33]. The buffer conditions were A1—10 mM ammonium acetate, pH 3.5 (adjusted with acetic acid); B1—99.9% acetonitrile with 0.1% formic acid; A2—10 mM ammonium acetate, pH 7.5 (adjusted with ammonia solution); and B2—99.9% methanol with 0.1% formic acid. All buffers were prepared in LC-MS grade water and organic solvents.

A list of all metabolites, including multiple reaction monitoring (MRM) ion ratios, retention times, and Kyoto Encyclopedia of Genes and Genomes (KEGG) or Human Metabolome Database (HMDB) metabolite identifiers can be found in Table S6. Peak integration was performed using MultiQuant software v.2.1.1 (Sciex, Foster City, CA) without any smoothing and reviewed manually. Peak intensities were normalized, first against the internal standards, and subsequently against protein abundances obtained from the BCA assay. The first transition of each metabolite was used for relative quantification between samples and controls. All original LC-MS-generated QTrap wiff- files, as well as MultiQuant processed peak integration q.session files can be downloaded via http://www.peptideatlas.org/PASS/PASS01195.

### 2.6. Proteomics Sample Preparation with Label-Free Quantification (LFQ)

Proteomics sample preparation was done according to a published protocol with minor modifications [34]. Three biological replicates of each patient and control fibroblast cell lines between the passages of 8–11 were harvested from T75 flasks and lysed under denaturing conditions in a buffer containing 6 M guanidinium chloride (GdmCl), 5 mM tris(2-carboxyethyl)phosphine, 20 mM chloroacetamide, and 50 mM Tris-HCl pH 8.5. Lysates were denatured at 95 °C for 15 min shaking at 800 rpm in a thermal shaker and sonicated in a water bath for 15 min. A small aliquot of cell lysate was used for the BCA assay to quantify the protein concentration. Lysates (100 µg proteins) were diluted with a dilution buffer containing 10% acetonitrile and 25 mM Tris-HCl, pH 8.5, to reach a 1 M GdmCl concentration. Then, proteins were digested with 2 µg LysC (MS-grade, Roche, enzyme to protein ratio 1:50) shaking at 800 rpm at 25 °C for 2 h. The digestion mixture was diluted again with the same dilution buffer to reach 0.5 M GdmCl. Then, 2 µg trypsin (MS-grade, Roche, enzyme to protein ratio 1:50) was added and the digestion mixture was incubated at 37 °C overnight in a thermal shaker at 800 rpm for 14 h. Solid phase extraction (SPE) disc cartridges (C18-SD, Waters, Milford, MA) were used for peptide desalting according to the manufacturer’s instructions. Desalted peptides were reconstituted in 0.1% formic acid in water and further separated into four fractions by strong cation exchange chromatography (SCX, 3M Purification, Meriden, CT). Eluates were first dried in a SpeedVac, then dissolved in 20 µL 5% acetonitrile and 2% formic acid in water, briefly vortexed, and sonicated in a water bath for 30 seconds prior injection to nano-LC-MS.

### 2.7. LC-MS Instrument Settings for Shotgun Proteome Profiling and Data Analysis

LC-MS/MS was carried out by nanoflow reverse-phase liquid chromatography (Dionex Ultimate 3000, Thermo) coupled online to a Q-Exactive Plus Orbitrap mass spectrometer (Thermo). Briefly, the LC separation was performed using a PicoFrit analytical column (75 μm ID × 55 cm long, 15 µm Tip ID; New Objectives, Woburn, MA) in-house packed with 2.1 µm C18 resin (Reprosil-AQ Pur, Dr. Maisch, Ammerbuch, Germany). Peptides were eluted using a non-linear gradient from 2% to 40% solvent B over 101 min at a flow rate of 266 nL/min (solvent A: 99.9% water, 0.1% formic acid; solvent B: 79.9% acetonitrile, 20% water, 0.1% formic acid). 3.5 kilovolts were applied for nanoelectrospray ionization. A cycle of one full fourier transformation scan mass spectrum (300−1750 *m*/*z*, resolution of 60,000 at *m*/*z* 200, automatic gain control (AGC) target 1 × 10^6^) was followed by 12 data-dependent MS/MS scans (200–2000 *m*/*z*, resolution of 30,000, AGC target 5 × 10^5^, isolation window 2 *m*/*z*) with normalized collision energy of 25 eV. Target ions already selected for MS/MS were dynamically excluded for 15 s. In addition, only peptide charge states between two to eight were allowed.

Raw MS data were processed with MaxQuant software (v1.6.0.1) and searched against the human proteome database UniProtKB with 21,074 entries, released in December 2018. Parameters of MaxQuant database searching were a false discovery rate (FDR) of 0.01 for proteins and peptides, a minimum peptide length of seven amino acids, a first search mass tolerance for peptides of 20 ppm and a main search tolerance of 4.5 ppm, and using the function “match between runs”. A maximum of two missed cleavages was allowed for the tryptic digest. Cysteine carbamidomethylation was set as fixed modification, while N-terminal acetylation and methionine oxidation were set as variable modifications. Contaminants, as well as proteins identified by site modification and proteins derived from the reversed part of the decoy database, were strictly excluded from further analysis. The MaxQuant processed output files can be found in Table S2, showing peptide and protein identification, accession numbers, percentage of sequence coverage of the protein, q-values, and LFQ intensities. The mass spectrometry data have been deposited to the ProteomeXchange Consortium (http://proteomecentral.proteomexchange.org) via the PRIDE partner repository [35] with the dataset identifier PXD009743.

### 2.8. Experimental Design, Statistical Rationale, Pathway, and Data Analyses

The correlation analysis of biological replicates and the calculation of significantly different metabolites and proteins were done with Perseus (v1.6.0.2). LFQ intensities, originating from at least two different peptides per protein group, were transformed by log_2_. Only protein groups with valid values within compared experiments were used for further data evaluation. Statistical analysis was done by a two-sample *t*-tests with Benjamini–Hochberg (BH, FDR of 0.05) correction for multiple testing. Significantly regulated metabolites and proteins between patients and controls were indicated by a plus sign in Tables S1 and S3.

One-way ANOVA with Tukey’s multiple comparison test (significance level, alpha = 0.05) was performed using GraphPad Prism 5 to compare concentration ratios of reduced and oxidized glutathione (GSH/GSSG) in patients and controls, as well as data from live cell respiration assay.

For comprehensive proteome data analyses, we applied gene set enrichment analysis (GSEA, v2.2.3) [36] in order to see if a priori defined sets of proteins showed statistically significant, concordant differences between mutations and controls. All proteins with ratios calculated by Perseus were used for GSEA analysis. The Galaxy online tool (https://usegalaxy.org/) was used to calculate the average of the ratios of the few duplicate gene names. We used GSEA standard settings, except that the minimum size exclusion was set to 5 and Reactome v5.2 and KEGG v5.2 were used as gene set databases. The cutoff for significantly regulated pathways was set to be *p*-value ≤ 0.05 and FDR ≤ 0.05.

### 2.9. Simulations of Electron Transfer between the Iron–Sulfur Clusters of NDUFS1 and Prediction of Protein Stability of p.Phe124Leu in ND5

We have applied tunneling calculations of electron transfer between N4 and N5 iron–sulfur clusters of the NDUFS1 subunit and studied the consequences of mutations of the key residues involved in the process using a method described previously [37].

The change of the Gibbs free-energy gap, ΔΔG, which measures the gain or loss of protein stability upon mutations, was calculated by the online tool STRUM for the p.Phe124Leu substitution in ND5 [38].

### 2.10. Measurement of Respiratory Chain Enzyme Activities 

Sample preparation of muscle homogenates for the spectrophotometric assay of enzyme activities was done as reported previously [39,40,41].

### 2.11. Live Cell Respiration Assay by Seahorse XFe96 

The operation and the calibration of sensor cartridges of the Seahorse XFe96 instrument were done according to the manufacturer’s instructions. A pilot experiment was performed to optimize the cell number at seeding and the concentration of carbonyl cyanide-4-(trifluoromethoxy)phenylhydrazone (FCCP), an uncoupling agent of the mitochondrial electron transfer chain and the ATP synthase. In the final assay, fibroblasts were seeded at 40,000 cells per well and cultured under normoxic condition (5% CO_2_) for 6 h in order to allow them to attach to the bottom of the culture plate. Then, the culture medium was replaced with the Seahorse assay medium and the plate was transferred to a non-CO_2_ incubator for 45 min right before the start of the assay. The Mito Stress Test Kit (Agilent, #103015-100) and the Glycolytic Rate Assay Kit (Agilent, #103344-100) were used according to the user manuals. Inhibitor concentrations were used as followed: oligomycin (2 µM), FCCP (1.5 µM), rotenone (0.5 µM), antimycin (0.5 µM), and 2-deoxy-glucose (500 mM).

### 2.12. Blue Native PAGE, Western Blot, and In-Gel Activity Assay of CI

Fibroblasts were trypsinized, collected by centrifugation, and lysed in mitochondria isolation and storage buffer (83 mM sucrose, 3.3 mM Tris-HCl, pH 7.0, 0.3 mM ethylenediaminetetraacetic acid (EDTA), 1.7 mM 6-aminohexanoic acid, and protease inhibitor cocktails) by passing them through a needle (Ø 0.45 × 25 mm, 26 G × 1") 30 times on ice, on the basis of published protocols with minor modifications [42,43]. In brief, crude mitochondria fractions were solubilized with digitonin in a ratio of 10 µL 20% digitonin per 20 mg cell pellet. Next, 8 µg solubilized mitochondria per lane were loaded onto the precast NativePAGE 3%–12% gradient Bis-Tris protein gels (Thermo, # BN1001BOX) and run at 4 °C with pre-chilled buffers. The blue native PAGE was first run with the dark blue cathode buffer (0.02 % Coomassie Blue G-250) until the running front reached one-third of the gel length. Then, the light blue cathode buffer (0.002% Coomassie Blue G-250) was used to finish the gel running. The gel was further processed for western blot or in-gel activity assay of CI. 

Immunodetection of OXPHOS enzymes on blue native gels was performed following an established protocol [43]. The antibodies used in western blot were anti-NDUFS1 (Proteintech, Rosemont, IL, USA, # 12444-1-AP) and all others were purchased from Merck: anti-NDUFS2 (# SAB2702088), anti-NDUFB8 (# HPA003886), anti-UQCRC2 (# HPA007998), and anti-SDHB (# HPA002868).

In-gel activity assays of CI were conducted as described previously [43]. Briefly, blue native gels were incubated in the assay buffer directly after electrophoresis. One half of the gel piece was cut out and incubated in the assay buffer for 1 hour and documented. The other half, as an identical replicate, was stained with Coomassie G-250 following a published protocol [44].

### 2.13. Protein Sequence Alignment

Protein sequences of NDUFS1 and ND5 were aligned using T-Coffee [45] and visualized in Espript 3.0 [46].

## 3. Results

### 3.1. Substituted Amino Acids in ND5 and NDUFS1 are Highly Conserved from Bacteria to Human

A protein sequence alignment across multiple species showed a highly conserved phenylalanine at position 124 in the subunit ND5, which was substituted to leucine in one patient (Figure S1A). The heteroplasmy level of this mutation was 70% in cultivated skin fibroblasts, which is in agreement with other reports, displaying a very severe phenotype [25,26,27]. Furthermore, Phe124 is localized in the fourth transmembrane helix of subunit ND5, which is close to the proposed proton translocation channel [27] and thus may influence its structure and catalytic function. In the other patient, two highly conserved amino acids, valine at position 228 and aspartate at position 252, were substituted to alanine and glycine in NDUFS1, respectively (Figure S1B). Val228 is located between the two iron–sulfur clusters N4 and N5 in subunit NDUFS1 (protein data bank, PDB: 5XTD, human CI).

### 3.2. Metabolome Profiling Revealed a Decrease of the GSH/GSSG Ratio in Both Patients

**Figure 1:**
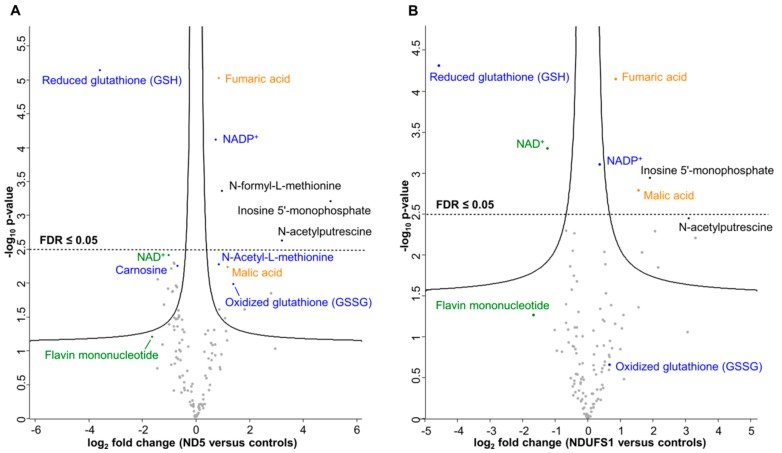
Significantly regulated metabolites between mutated *MT-ND5* and *NDUFS1* versus controls, respectively. (**A**) Abundance ratios of metabolites between mutated *MT-ND5* and controls. (**B**) Abundance ratios of metabolites between mutated *NDUFS1* and controls. Metabolites above the solid lines were considered significant after the *t*-test. Metabolites above the dashed horizontal line were significant after Benjamini–Hochberg correction (false discovery rate (FDR) ≤0.05) for multiple testing. Log_2_ fold changes were plotted against the -Log10 (*p*-value). Blue: metabolites involved in cellular oxidative stress response; orange: metabolites of the TCA cycle; green: CI-related metabolites.

To quantify relative differences in metabolite changes and to elucidate key metabolic alterations caused by mutating the *MT-ND5* and *NDUFS1* genes, we applied a targeted liquid chromatography-mass spectrometry (LC-MS/MS) approach based on multiple reaction monitoring (MRM) [33]. In total, 121 metabolites were quantified relatively (Table S1). The Pearson correlation coefficients were highly similar, ranging from 0.968 to 0.996 in the controls, 0.984 to 0.996 in the *MT-ND5* mutation, and 0.985 to 0.991 in the *NDUFS1* mutations (Figure S2), suggesting a very good quality of the metabolite data sets. Statistical analysis by an unpaired two-sample *t*-test identified significantly regulated metabolites, and six of them were significant after Benjamini–Hochberg (BH, FDR ≤ 0.05) correction for multiple testing (*p*-value ≤ 0.05) in the *MT-ND5* mutation versus controls (Figure 1A). In the case of the *NDUFS1* mutations, 11 significant metabolites were found after the *t*-test and six were identified upon BH correction (*p*-value ≤ 0.05, FDR ≤ 0.05) (Figure 1B). Interestingly, the same metabolites were found to be significantly regulated in both patients.

**Figure 2:**
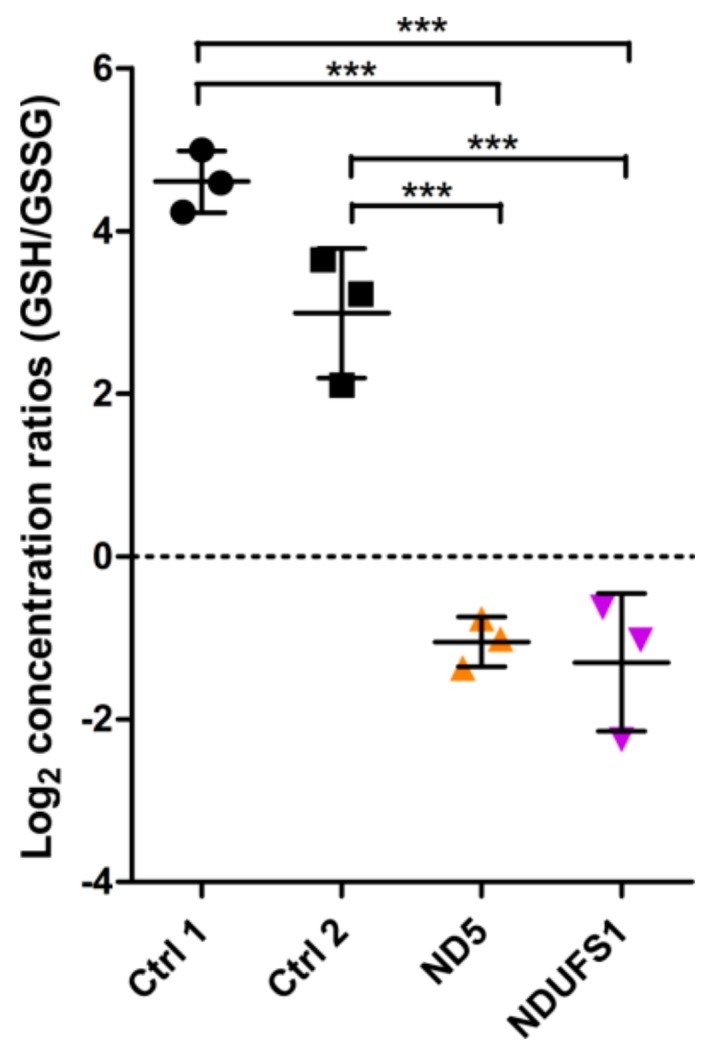
Reduced and oxidized glutathione (GSH/GSSG) ratios of controls, ND5, and NDUFS1 mutant fibroblast cells. The log_2_ concentration ratios (GSH/GSSG) were compared between patients and controls. One-way ANOVA of log_2_ ratios was performed (*p*-value < 0.001, labeled as ***). Error bars: mean ± SD.

Glutathione (GSH) was the metabolite with the highest decrease in both patients (12-fold in ND5, 16-fold in NDUFS1, Figure 1A,B). In contrast, oxidized glutathione (GSSG) levels were increased in both patients. The ratio between reduced and oxidized glutathione (GSH/GSSG ratio) can be used as a marker for the redox status of a cell [47,48,49]. The concentration ratios of GSH/GSSG between patients and controls decreased significantly (more than 35-fold) for both patients’ fibroblast cells (Figure 2), thus indicating a higher level of oxidative stress. Furthermore, the polyamine N-acetylputerescine was significantly increased in both patients, also indicative for higher stress levels [50,51].

### 3.3. The TCA Cycle Metabolites—Fumaric and Malic Acid—Significantly Increased in Both Patients

Malate and fumarate are two TCA cycle intermediates that were significantly upregulated in both patients. Similar findings have been reported in patients’ urine samples [52]. Lactic acid, which is converted from pyruvate, was elevated twofold in both patients. This is in concordance with frequently observed lactic acidosis in patient blood [53]. The ability of CI to oxidize NADH in both patients seems to be limited and, as a consequence, the level of NAD^+^ was found to be significantly reduced (Figure 1A,B).

Furthermore, flavin mononucleotide (FMN) and riboflavin (vitamin B2, a precursor of FMN) were both decreased threefold in patient fibroblasts carrying the *MT-ND5* mutation. FMN is a prosthetic group of mitochondrial CI and accepts electrons from NADH.

The metabolome survey identified that the same metabolites were significantly regulated in both patients. These metabolites, such as GSH, GSSG, NAD^+^, NADP^+^, FMN, malate, and fumarate, are all directly or indirectly involved in, or dependent on the functionality of CI.

### 3.4. Proteome Profiling 

Fibroblasts harboring the *MT-ND5* and *NDUFS1* mutations were compared to two individual healthy controls in triplicates by label-free quantification (LFQ) in a total of 60 LC-MS/MS runs. We identified more than 5363 protein groups with at least two peptides per protein group (Table S2). We then compared both mutations individually to controls and filtered for 100% valid values in at least one group and replaced missing values from the normal distribution. This resulted in a total of 4030 protein groups for the *MT-ND5* patient and 3893 for the *NDUFS1* patient versus controls (Table S3). 

The reproducibility of the biological replicates was tested by Pearson correlation and visualized in a multi-scatter plot for all experiments. The Pearson correlation coefficients were highly similar, ranging from 0.959 to 0.994 in controls, 0.989 to 0.992 in the *MT-ND5* mutation, and 0.988 to 0.994 in the *NDUFS1* biallelic mutations (Figure S3), indicating very robust replicates.

**Figure 3:**
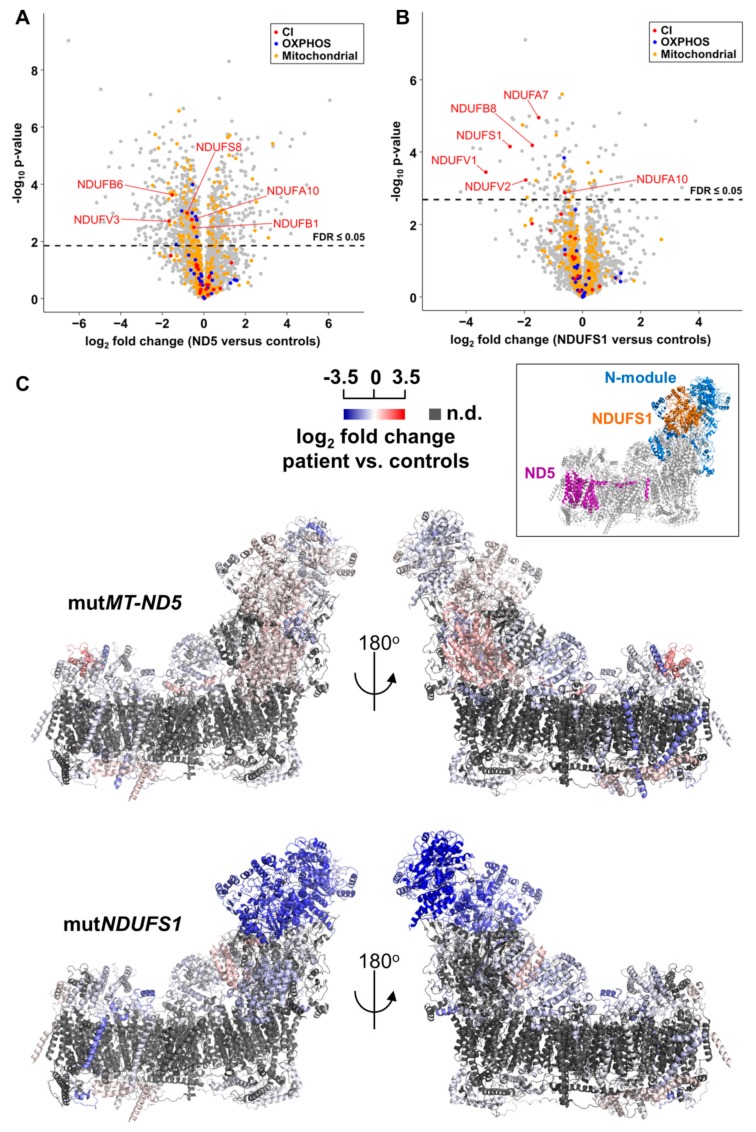
Abundance ratios of CI subunits mapped onto the CryoEM structure of human CI, PDB: 5XTD. (**A**) Protein abundance ratios between mutND5 and controls. Mitochondrial proteins according to Human MitoCarta2.0 are shown in orange, OXPHOS proteins in blue, and CI subunits in red. The dotted line indicated the threshold of significance (FDR ≤0.05) in the two-sample *t*-test after Benjamini–Hochberg correction. Protein names are presented for significantly regulated CI subunits. (**B**) Protein abundance ratios between mutNDUFS1 and controls. Same legend as in (**A**). (**C**) The mutation in *MT-ND5* did not lead to a general change of the abundance of CI subunits, but a specific loss of the N-module was identified in the *NDUFS1* patient. The inset indicates the relative position of the N-module, NDUFS1, and ND5 in CI. n.d., no data.

Statistical analysis by an unpaired two-sample *t*-test identified 1535 significant proteins and 1090 significantly regulated proteins after Benjamini–Hochberg (BH) correction for multiple testing (*p*-value ≤ 0.05, FDR ≤ 0.05) in the *MT-ND5* mutation versus controls, as visualized in a volcano plot (Figure 3A). In the case of the *NDUFS1* mutations, 324 significantly deregulated proteins were found after the *t*-test, and 145 were identified upon BH correction (*p*-value ≤ 0.05, FDR ≤ 0.05) (Figure 3B).

### 3.5. Gene Set Enrichment Analyses Reveal Glycolysis is Upregulated in the MT-ND5 Mutation and the Respiratory Chain is Down-Regulated in the NDUFS1 Mutations 

We applied the pathway enrichment tool GSEA to assess whether a priori defined sets of proteins showed statistically significant, concordant differences between *MT-ND5* and *NDUFS1* versus controls, respectively. Pathways with significant *p*-values (≤0.05) and FDR q-values (≤0.05) are listed in Tables S4 and S5. In the patient with the *MT-ND5* mutation, pathways comprising proteins of the cytoskeleton, the extracellular matrix, cytosolic tRNA aminoacylation, and glycolysis and gluconeogenesis were significantly upregulated (Table S4).

Many structural and cytoskeleton proteins were enriched in the *MT-ND5* patient, such as myosins MYL9 and MYLK, actin ACTA2, tropomyosins, TPM1, CALD1, MYL6, TPM4, FN1, and collagens, reflected in the respective pathways of muscle contraction, focal adhesion, and collagen formation.

The pathway “cell cycle” was significantly down-regulated in cells harboring the *MT-ND5* mutation, including six of the proteins of the mini-chromosome maintenance complex (MCM) responsible for DNA replication, (threefold, see Table S4).

In the case of the patient with *NDUFS1* mutations, the pathway including cytoskeleton components was only significantly increased at a nominal *p*-value (Table S5).

The respiratory electron chain was the only significantly down-regulated pathway in the patient carrying *NDUFS1* mutations (Table S5). To further shed light on the substructures of CI, we performed a pathway analysis with manually created gene lists for all modules of CI. This analysis identified a significant and specific decrease only in the N-module (*p* ≤ 0.001, q-value ≤0.05, Table S5), including the subunits NDUFA7 (threefold), NDUFV2 (fourfold), NDUFS1 (sixfold), and NDUFV1 (tenfold) (Figure 3B).

To visualize this dysregulation, all protein abundance ratios of CI subunits between patients and controls were mapped in a three dimensional structure of CI (PDB: 5XTD). The inlet indicated the position of subunit ND5 and NDUFS1 in CI (Figure 3C). Indeed, the N-module in the *NDUFS1* patient was the only region to be severely reduced, whereas the *MT-ND5* patient showed no changes (Figure 3C). This indicated that the stability of the entire N-module was affected, most likely because of fast degradation of misfolded or not integrated subunits of CI.

### 3.6. The Rate of Electron Tunneling between the Iron–Sulfur Clusters N4 and N5 of NDUFS1 Was Predicted to Decrease Dramatically in a V228A Mutant

**Figure 4:**
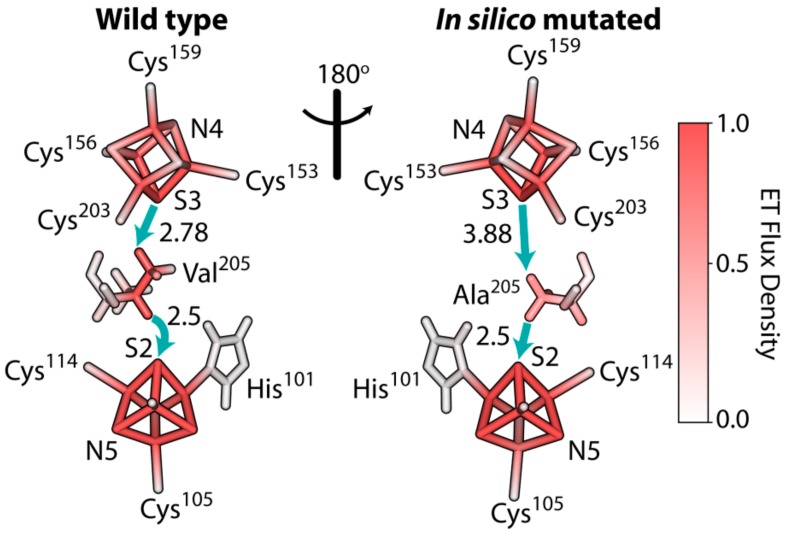
Electron tunneling pathway of the electron transfer reaction between iron–sulfur clusters N4→N5 of the wild-type and *in silico* mutated CI in *Ovis aries*, PDB 5LNK. Solid blue arrows indicate through-space jumps in the primary electron tunneling pathways. Through-space distances in Ångstrom are shown next to the arrows. Relative color density indicates the contribution of the corresponding atom/bond in electron transfer reaction. The mutation resulted in a reduced rate of electron transfer $k_{ET}^{WT}/k_{ET}^{V205A}=\;35$ for the ovine enzyme.

We examined the electron transfer between the iron–sulfur clusters N4 and N5 in subunit NDUFS1 using the method of tunneling current theory, as was previously described for a bacterial enzyme [37]. It revealed that the residue Val228 was critical for bridging the electron transfer between the N4 and N5 clusters, as electrons tunnelled primarily through this relatively bulky residue. Our simulations for an ovine enzyme showed that the mutation p.Val205Ala, with a smaller alanine substitution, had a dramatic effect on the rate of electron transfer by reducing it by 35-fold (Figure 4). It is interesting that the effect of mutation of this critical valine residue was predicted to occur earlier in *Thermus thermophilus* [37]. These changes were predicted to occur in the remaining small fraction of fully assembled CI.

### 3.7. Decreased Stability of CI in Mutated NDUFS1 Prevents the Formation of Supercomplexes 

**Figure 5:**
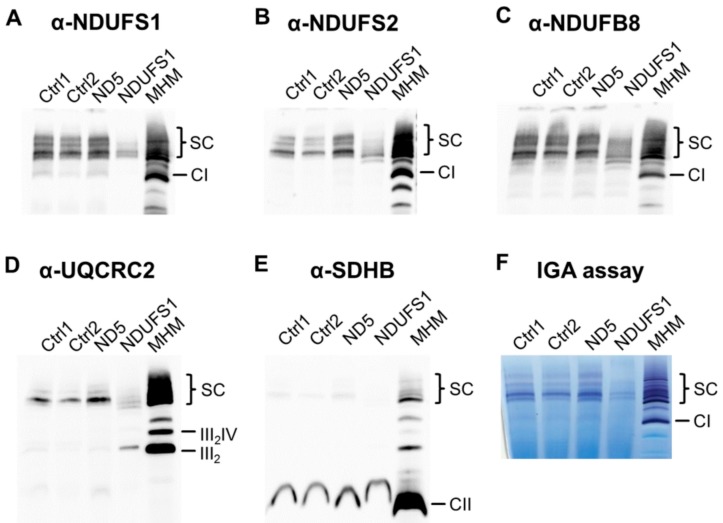
Formation of respiratory chain supercomplexes and in-gel activity assay of CI. Blue native PAGE and western blot detection of respiratory chain enzymes solubilized with digitonin. (**A**) Antibody against NDUFS1, a core subunit of the N-module in CI; (**B**) antibody against NDUFS2, a core subunit of the Q-module in CI; (**C**) antibody against NDUFB8, an accessary subunit of the P-module in CI; (**D**) antibody against UQCRC2, a core subunit of CIII; (**E**) antibody against SDHB, a subunit of complex II (CII), which serves as a loading control. (**F**) In-gel activity assay (IGA) of CI. Ctrl: control; MHM: mouse heart mitochondria, as molecular weight marker and positive control; SC: supercomplexes; III, complex III (CIII); IV, complex IV (CIV).

To elucidate the consequences of the specific loss of the N-module in the mutated *NDUFS1* cell line for the formation of supercomplexes, we solubilized OXPHOS proteins under mild conditions using digitonin, and performed blue native PAGE followed by western blot. Only a small fraction of supercomplexes were formed compared to controls (Figure 5A–D). In addition, a major part of CIII was not incorporated into supercomplexes, which was the stoichiometric assembly of CI, III, and IV (Figure 5D). In contrast, no assembly errors were identified in the *MT-ND5* patient. Succinate dehydrogenase (complex II, CII) was used as a loading control and showed no differences between samples (Figure 5E).

To test the functionality of the partly assembled respirasome, an in-gel activity assay of CI was performed and revealed that there was almost no enzymatic activity in the patient with *NDUFS1* mutations (Figure 5F).

### 3.8. Isolated CI Deficiency in Both Patients

Enzymatic measurements of the respiratory chain enzymes and citrate synthase (CS) were performed in the homogenates of muscle biopsies for both patients [40,54]. These were normalized to CS and were compared to reference values (Table 1) [31]. In both cases, the CI enzyme activity was below the reference values, while all other complexes showed values within the range of the references, indicating an isolated CI deficiency.

### 3.9. Live Cell Respiration Assays Revealed a Low Oxygen Consumption Rate in Both Patients

**Figure 6:**
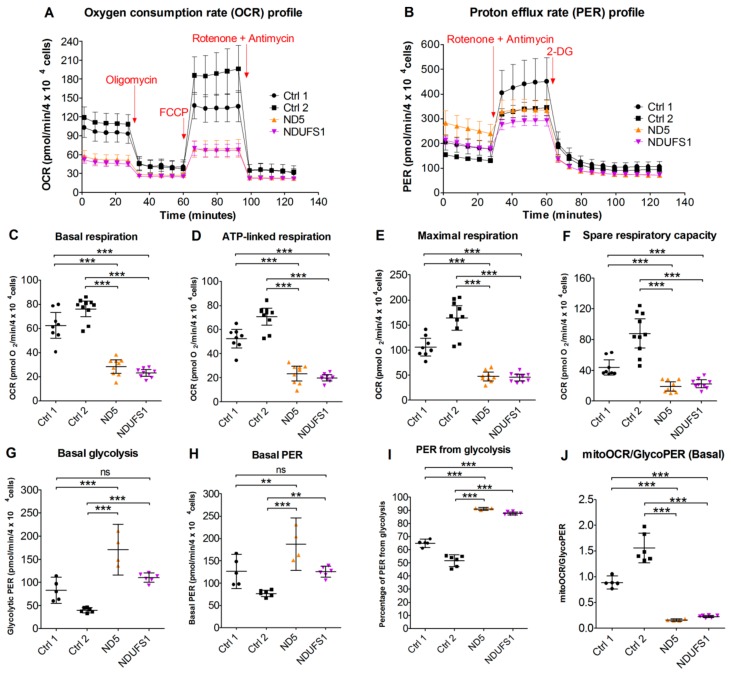
Cellular respiration assays showing mitochondrial dysfunction and increased glycolytic activities in both patients. (**A**) Oxygen consumption rate profile. (**B**) Proton efflux rate (PER) profile. (**C**) Basal respiration rate at the beginning of the assay. (**D**) ATP-linked respiration before the addition of the inhibitors. (**E**) Maximal respiration after carbonyl cyanide-4-(trifluoromethoxy)phenylhydrazone (FCCP) was added. (**F**) Calculated spare respiratory capacity. (**G**) Basal glycolysis rate at the beginning of the assay. (**H**) Total PER before the addition of the inhibitors. (**I**) Percentage of PER from glycolysis. (**J**) The ratio between mitochondrial oxygen consumption rate (OCR) and glycolytic PER as an indicator of the cellular energetic profile. Error bars: (**A**) and (**B**): mean ± SD; (**C**–**J**): mean ± 95% confidence intervals. One-way ANOVA: *p*-value < 0.01 (**); *p*-value < 0.001 (***); ns, not significant.

The respiration rate measurements in live cells (Figure 6A) elucidated that the basal respiration, ATP-linked respiration, maximal respiration, and the spare respiration capacity were less than 50% compared with controls (Figure 6C–F). In contrast, the basal glycolysis rate was significantly higher (*p*-value ≤ 0.05) in patients than in the controls (Figure 6G). The glycoPER (Figure 6B), which is the PER (proton efflux rate) contributed by glycolysis, was significantly higher (*p*-value ≤ 0.05) in the patients (Figure 6H,I). Thus, a clear metabolic shift from aerobic respiration to glycolysis was observed in both mutated cell lines (Figure 6J), which is in concordance with our proteomics data for the patient with the *MT-ND5* mutation, where the glycolytic pathway was upregulated.

## 4. Discussion

Mitochondrial dysfunction is the most common type of metabolic disorder and can be caused by either mitochondrial or nuclear gene mutations. Here, we applied proteome and metabolome profiling to reveal the molecular consequences of gene mutations in *NDUFS1* and *MT-ND5*, which respectively encode for the two core subunits in the hydrophilic and transmembrane arms of CI.

### 4.1. Specific Disassembly of the N-Module and the Entire Respirasome in Mutated NDUFS1

Our proteome screening indicated a specific loss of the entire N-module for the *NDUFS1* patient (Figure 3C). For validation, we applied BN-PAGE in combination with western blot to reveal a disrupted assembly for CI. An in-gel activity assay showed a missing band only in this patient, which may either derive from a diminished stability of CI or by the lack of FMN in the protein (Figure 5F). The CI N-module consisted of three core subunits, NDUFS1, NDUFV1, and NDUFV2, which were encoded by nuclear genes and accommodate the FMN prosthetic group, as well as the iron–sulfur clusters N1a, N3, N1b, N4, and N5 [55,56]. The N-module in the patient with mutated *NDUFS1* hence disintegrated easily, resulting in strongly reduced amounts of fully assembled CI, causative for to the observed enzymatic dysfunction. The homozygous mutation p.Asp252Gly in NDUFS1 alone was shown to cause the disassembly of CI in a patient with mild cavitating leukoencephalopathy [30]. Recent studies support a model that functional modules of CI are first assembled independently and then gradually form a mature CI, in which the N-module joins in the final step [57,58,59]. Over the past two decades, several possible formations of structures for respiratory chain supercomplexes have been identified and resolved at high resolution [60,61,62,63,64,65,66]. It has been reported that about 80–90% of the CI population is indeed bound to other OXPHOS complexes in stoichiometry to form supercomplexes (SC), which is named the mitochondrial respirasome and is composed of CI, CIII and CIV [67,68,69]. The formation of SC was severely reduced in the *NDUFS1* patient compared with the controls (Figure 5A–D). This was further confirmed by the detection of large amounts of individual CIII, which could not be assembled into a mature SC in the *NDUFS1* patient (Figure 5D). Whether the formation of respirasomes indeed enhances the efficiency of electron transfer and minimizes the electron leakage and thus ROS production is still controversially debated [63,64,70,71]. It can be concluded that the N-module disintegrated easily due to the substitutions of two amino acids in the *NDUFS1* patient, which severely affected the maturation and structural stability of CI and, hence, the formation of SC.

### 4.2. Disruption of The Electron Flow in Mutated NDUFS1

In the patient with *NDUFS1* mutations, the valine at position 228 was changed to alanine. This valine was located between the iron–sulfur cluster N4 and N5 (Figure 4). A previous study modeled the effect of this Val232Gly substitution in bacterial CI and showed that the “Y”-shaped side chain of valine is crucial as a bridge for electron transfer between N4 and N5. The replacement of valine to glycine caused a decrease in the electron transfer efficiency to about one-thousandth in bacterial CI [37]. Our *in silico* modeling of the ovine enzyme with the specific substitution p.Val205Ala again confirmed the dramatically reduced electron transfer, which was expected in the *NDUFS1* patient. The decreased rate of electron transfer between the N4 and N5 clusters should affect the overall rate of electron transfer from NADH via FMN and iron–sulfur clusters to Co-Q10 in CI, with an elevated level of FMN in its reduced state and a consequential increase in the level of ROS production by the enzyme [72,73,74,75]. Therefore, both defects, the Val228Ala substitution disrupting electron transfer between the N4 and N5 clusters and the partial disintegration of the N-module due to the Asp252Gly substitution [30], might be the cause of the elevated ROS production, indicated by the decreased GSH/GSSG ratio in the patient (Figure 2). Elevated ROS has been reported in cells with CI assembly defects previously [76]. The reported case, carrying only the p.Asp252Gly substitution, presented a very mild phenotype [30], indicating that the additional interruption of the electron flow between N4 and N5 in our case significantly contributed to the severity of the phenotype.

Regarding tunneling calculations, however, several important points should be mentioned. The change of electron transfer coupling between N4 and N5 was quite significant in all enzymes that we have examined. However, given all the uncertainties in the structure, and difficulties of theoretical modeling of FeS clusters, these results should be regarded only as qualitative trends. It was also recognized that a slower electron transfer rate will only be important if this is the rate-determining step. Furthermore, we cannot exclude that additional water molecules will occupy the mutated site, possibly changing the rates of electron transfer. Generally, it should be recognized that the accurate quantitative predictions on human enzymes is still a significant challenge. However, the calculated dramatic disruption of electronic coupling reported here, and in the emerging picture, appears to be in agreement with overall experimental evidence collected in this work on the *NDUFS1* mutant.

### 4.3. The Stalling of Proton Translocation in Mutated ND5 Is Assumed to Stop Electron Flow Without Any Consequences for Respirasome Formation

In contrast, the other patient with the *MT-ND5* mutation, harboring a heteroplasmy level of 70%, had a fully assembled CI (Figure 5). The amino acid substitution in the ND5 subunit, which was located on the distal end of the transmembrane arm, thus had no effect on the assembly of CI. The crystal structure of CI suggests that a unique, out-of-the-membrane quinone-reaction chamber enables redox energy to drive concerted long-range conformational changes, resulting in the translocation of four protons upon oxidation of one NADH molecule [77,78,79]. A study in *Escherichia coli* showed that amino acid substitutions close to the proton translocation channel indeed reduced the functionality of CI [80]. Thus, we expect that the proton pumping activity in the *MT-ND5* patient to be impaired in a similar way. In silico modeling of this effect by the online tool STRUM, a structure-based prediction of protein stability changes upon single-point mutation [38], indeed showed increased stability for the p.Phe124Leu mutation in subunit ND5. Hence, the stabilizing effect of this mutation for the proton channel might hamper its functionality by losing its flexibility. However, the details of the conformational coupling to electron flow remain unknown. It is worth mentioning that the mutation in proton pumping regions hindering electron transfer far away is one of the marvels of CI, and this has been demonstrated by earlier experiments on isolated proteins. One can argue that if the *MT-ND5* mutation indeed results in disrupted conformational coupling and affects the electron transfer chain, the immediate consequence of this may be an elevated production of ROS, either by reduced FMN or by a reversed electron transfer mechanism [81,82,83,84].

### 4.4. A Similar Pattern of Regulated Metabolites Was Identified in Both Patients, Mainly for ROS Defense and TCA Cycle Metabolites

Interestingly, an almost identical set of significantly regulated metabolites was identified in both patients (Figure 1). The shortage of NAD^+^ may reflect the deficiency of CI, one of the major consumers that oxidizes NADH and generates NAD^+^. We further want to mention that an imbalance of the NADH/NAD^+^ ratio in itself may affect all aspects of impaired metabolism [85,86]. A stable NADH/NAD^+^ ratio is critical toward maintaining the homeostasis of metabolic process in both the cytoplasm and mitochondria [85,87]. An increased NADH/NAD^+^ ratio might thus affect the TCA cycle, since previous studies have shown that the inhibition of CI increased succinate oxidation rates [33,88]. The TCA cycle is primarily regulated by product feedback inhibition by NADH and by ADP/ATP and NAD^+^/NADH ratios [89]. The lack of NAD^+^, required for the conversion of malate to oxaloacetate, resulted in the elevated malate levels. Secondary metabolic alterations caused by CI deficiency were found previously in urine and may play an important role in the pathogenesis of CI deficiency [52]. Fumaric, malic, and also lactic acid, were found to be dramatically increased in some patients’ urine, matching our cell culture results [53]. We therefore believe that NADH cannot efficiently transfer electrons to CI, either because of disassembly or because of dramatic disruption of the electron tunneling flow, and thus a jam of unused NADH was created, leading to dysregulation of these TCA cycle metabolites.

The N-module of CI binds and oxidizes NADH and generates two electrons that are transferred through FMN and seven iron–sulfur clusters to ubiquinone in the Q-module [24]. The fundamental role of FMN for the enzymatic functionality of CI has been demonstrated for specific mutations within the FMN docking side carrying subunit NDUFV1. Mutants lacking FMN were fully assembled, but enzymatically inactive [90]. FMN was diminished threefold in both of our cases and previously in rotenone inhibited cells [33], indicating that the electron transfer to quinone was interrupted. It has been reported for both prokaryotes and eukaryotes that the non-covalently bonded prosthetic group FMN dissociated reversibly from CI when the later one was reduced by NADH and no suitable electron acceptor was available [91,92]. The dissociation of FMN was proposed as a protective mechanism to decrease ROS production [92], as FMN was shown to be a direct site for superoxide radical formation in the CI N-module [73,74,93,94].

Mitochondria are a major source for ROS [81], which can be eliminated by ROS scavengers such as glutathione (GSH) [95]. Apparently, the more than 35-fold decrease in the GSH/GSSG ratio observed in both patients (Figure 2) was a strong indicator of elevated ROS levels because of a stalled electron flow. In addition, N-acetylputerescine, the inactive form of puterescine, was significantly increased in both patients. Puterescine is known to be a main ROS scavenger as well [50,51].

### 4.5. CI Deficiency Leads to a Glycolytic Phenotype

The cell respiration assay confirmed that both patients were indeed more glycolytic in their bioenergetics profiles, compared with the two controls (Figure 6B). The oxygen consumption rate (OCR) linked with ATP synthesis showed severe decreases in both patients’ fibroblasts (Figure 6D), which confirmed the dysfunction of the mitochondrial OXPHOS caused by CI mutations. This was in agreement with the clinical data from patient muscle biopsies, in which the CI activities were found to be below the reference range and matched the elevated lactate levels in the fibroblasts and plasma samples of the patients (Table 1). Furthermore, the maximal and spare respiration capacities were significantly lower in both patients (Figure 6E,F). In order to compensate for the mitochondrial shortage of ATP, the patients’ cells exhibited an increased rate of glycolysis, as indicated by the glycolysis-contributed proton efflux rate measurement (glycoPER, Figure 6G).

### 4.6. Accumulation of Structural Proteins in Patients

An increase of proteins involved in the cytoskeleton and the extracellular matrix (ECM) was found in both patients, in agreement with the diagnosis of ventricular hypertrophic cardiomyopathy in the *MT-ND5* patient. CI deficiency frequently resulted in remodeling of the extracellular matrix, causing cardiomyopathy [96,97,98]. A relationship between a compromised respiratory chain and alterations in structural proteins has been shown previously [99], and OXPHOS deficiencies have also been linked to the development of hypertrophic cardiomyopathy [100]. Thus, our proteome survey indicated that an insufficient cellular bioenergetic status led to an increase in the ECM and cytoskeletal mass, but further studies are necessary to provide mechanistic links.

## 5. Conclusions

We have characterized the molecular consequences of two distinct CI mutations that result in the stalling of electron flow within CI by two different mechanisms. In the *NDUFS1* patient, destabilization of the N-module and, in addition, an interruption of electron tunneling between the iron–sulfur clusters N4–N5 of the remaining assembled CI was observed. In the *MT-ND5* patient, a dysfunctional proton channel might be less efficient to translocate protons utilizing the energy provided by the electron transfer. The interruption of the electron flow led to electron leakage and, in turn, to increased ROS generation, as seen by the reduced GSH/GSSG ratios in both cases. Furthermore, the isolated CI deficiency induced a metabolic switch towards a glycolytic phenotype, and the imbalance of the NADH/NAD^+^ ratio caused an identical feedback on the regulation of TCA cycle metabolites in both mutations.

## Figures and Tables

**Table 1:** Enzyme activities of oxidative phosphorylation (OXPHOS) complexes in muscle biopsies.

Activity Ratio Versus CS	CI	CI + III	CII	CII + III	CIII	CIV	F_1_F_o_ ATP Synthase
Patient *MT-ND5*	0.04	0.07	0.29	0.39	2.10	1.87	0.96
Patient *NDUFS1*	0.05	0.14	0.29	0.36	2.20	0.97	1.16
Reference range	0.14–0.35	0.24–0.81	0.23–0.41	0.30–0.67	1.45–3.76	0.82–2.04	0.42–1.26

Clinical reference ranges are referred to a previous publication [31]. CS: citrate synthase, C: complex. Enzyme-specific activities were expressed as nanomoles of substrate per minute per milligram of protein (nmol/min/mg protein) and were normalized to the enzyme activity of CS. Indicated are ratios.

## Supplementary Materials

The following are available online at https://www.mdpi.com/2073-4409/8/10/1149/s1, Figure S1: Protein sequence alignment of ND5 and NDUFS1. Mutated amino acids in patients were indicated with red triangles. Secondary structure elements are shown above the alignments. (**A**) Sequence alignments for ND5. (**B**) Sequence alignments for NDUFS1. Figure S2: Pearson correlation of MRM-targeted metabolome data among patients and controls. Correlation coefficients are shown in the squares. Ctrl, control; rep, replicate. Figure S3: Pearson correlation of label-free quantification (LFQ) proteome data among patients and controls. Correlation coefficients are shown in the squares. Ctrl, control; rep, replicate. Table S1: Identified metabolite ratios between patients and controls. Fold changes for each metabolite and Benjamini–Hochberg (BH) corrected two-sample *t*-test values are indicated. Table S2: MaxQuant output file featuring the proteome profiles of fibroblasts harboring the *MT-ND5* and *NDUFS1* mutations, as well as healthy controls with LFQ intensities. Table S3: Proteome analysis by Perseus with fold changes between mutations versus controls and two-sample *t*-test significances. Table S4: Gene set enrichment analysis (GSEA) report of all upregulated (sheet 1) and downregulated (sheet 2) pathways in the patient carrying the *MT-ND5* mutation. The significant threshold was set to *p*-value ≤ 0.05, FDR ≤0.05, and is highlighted in green. Table S5: GSEA report of all upregulated (sheet 1) and downregulated (sheet 2) pathways in the patient carrying the *NDUFS1* mutations. The significant threshold was set to *p*-value ≤ 0.05, FDR ≤0.05, and is highlighted in green. Table S6: Mass spectrometry transition settings for metabolites and MRM ion ratios. RT of 0 min indicates that the metabolite was measured continuously because of the long elution time of the compound.
