# Supplementary material for: Mutations in NDUFS1 Cause Metabolic Reprogramming and Disruption of the Electron Transfer
Source: Cells. 2019 Sep 25;8(10):1149. doi: 10.3390/cells8101149 (PMC6829531; doi:10.3390/cells8101149)
Supplement: Supplementary file 1 [file cells-08-01149-s001.zip › Supplemental files.docx]

**Supplemental files**

**Supplemental Figure S1. Protein sequence alignment of ND5 and NDUFS1. Mutated amino acids in patients were indicated with red triangles.** Secondary structure elements are shown above the alignments. (A) Sequence alignments for ND5. (B) Sequence alignments for NDUFS1.

**Supplemental Figure S2. Pearson correlation of MRM targeted metabolome data among patients and controls.** Correlation coefficients are shown in the squares. Ctrl, control; rep, replicate.

**Supplemental Figure S3. Pearson correlation of LFQ proteome data among patients and controls.** Correlation coefficients are shown in the squares. Ctrl, control; rep, replicate

**Supplemental Table S1.**

**Identified metabolite ratios between patients and controls.** Fold changes for each metabolite and BH corrected two-sample t-test values are indicated.

**Supplemental Table S2.**

**MaxQuant output file featuring the proteome profiles of fibroblasts harboring the MT-ND5 and NDUFS1 mutations as well as healthy controls with LFQ intensities.**

**Supplemental Table S3.**

**Proteome analysis by Perseus with fold changes between mutations versus controls and two-sample t-test significances.**

**Supplemental Table S4.**

**GSEA report of all upregulated (sheet 1) and downregulated (sheet 2) pathways in the patient carrying the MT-ND5 mutation.** The significant threshold was set to p-value ≤0.05, FDR ≤0.05 and is highlighted in green.

**Supplemental Table S5.**

**GSEA report of all upregulated (sheet 1) and downregulated (sheet 2) pathways in the patient carrying the NDUFS1 mutation.** The significant threshold was set to p-value ≤0.05, FDR ≤0.05 and is highlighted in green.

**Supplemental Table S6.**

**Mass spectrometry transition settings for metabolites and MRM ion ratios.** RT of 0 min indicates that the metabolite was measured continuously, due to the long elution time of the compound.
