# Supplementary figures and images for: Mutations in NDUFS1 Cause Metabolic Reprogramming and Disruption of the Electron Transfer
Source: Cells. 2019 Sep 25;8(10):1149. doi: 10.3390/cells8101149 (PMC6829531; doi:10.3390/cells8101149)

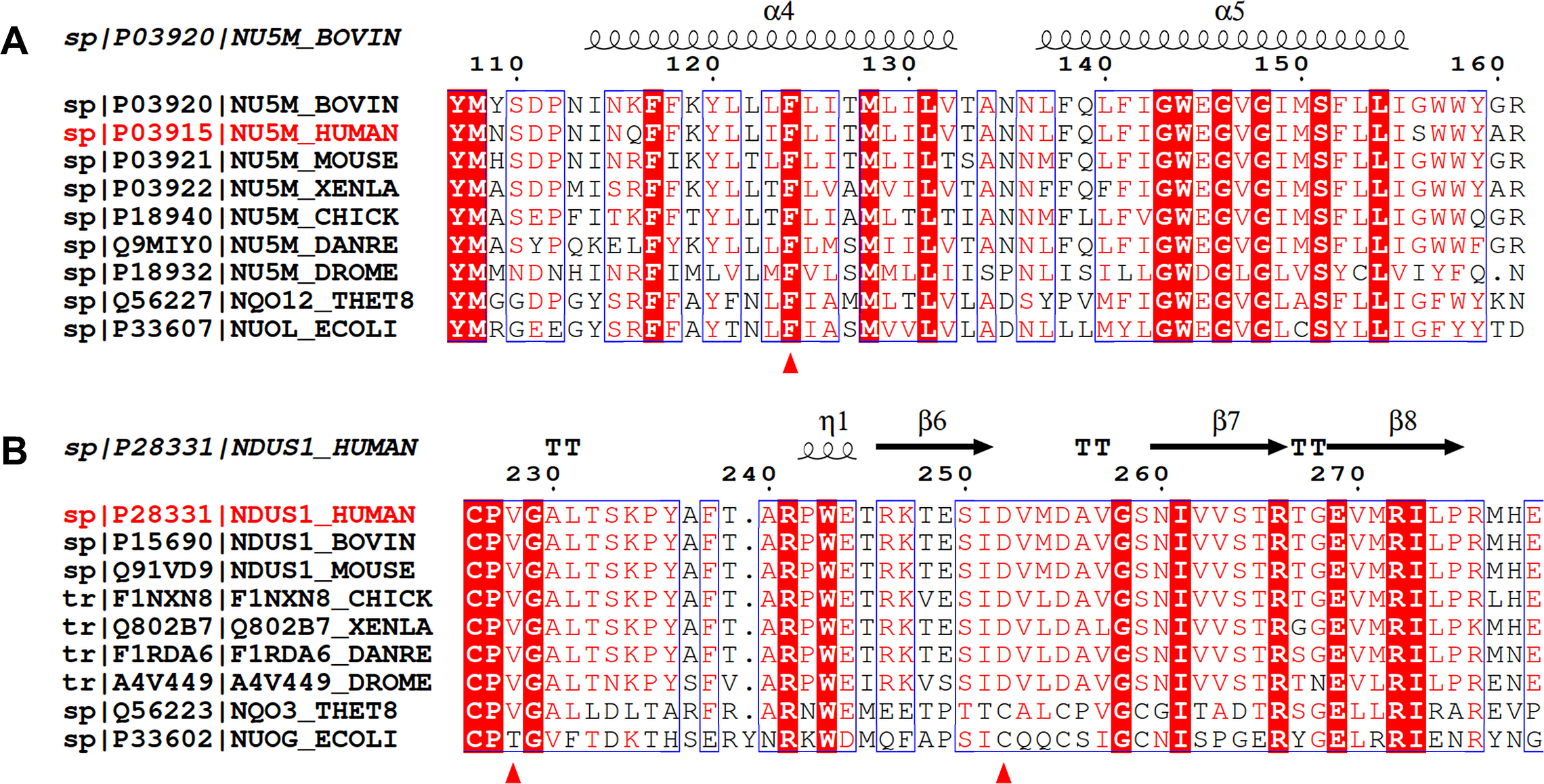

Supplement: Supplementary file 1 [file cells-08-01149-s001.zip › Supplemental Figure S1.tif]

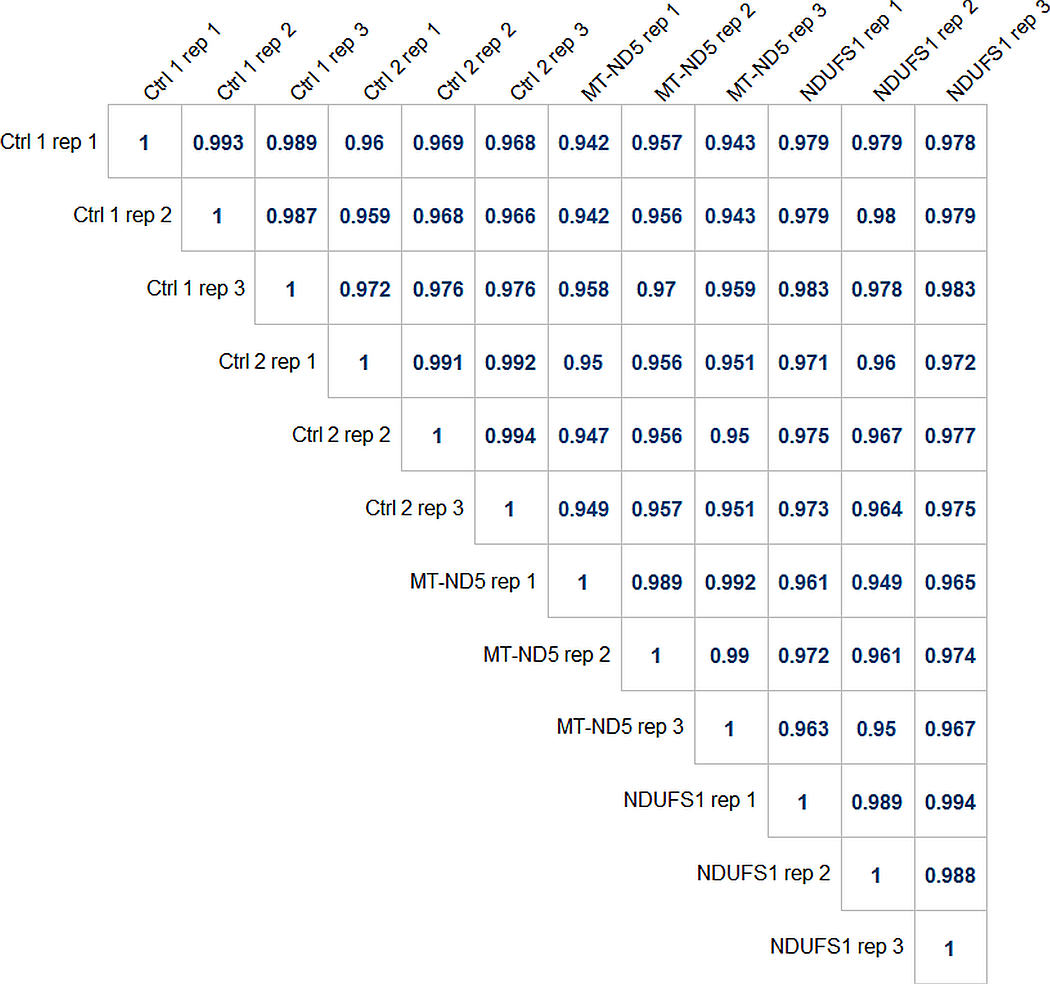

Supplement: Supplementary file 1 [file cells-08-01149-s001.zip › Supplemental Figure S2.tif]

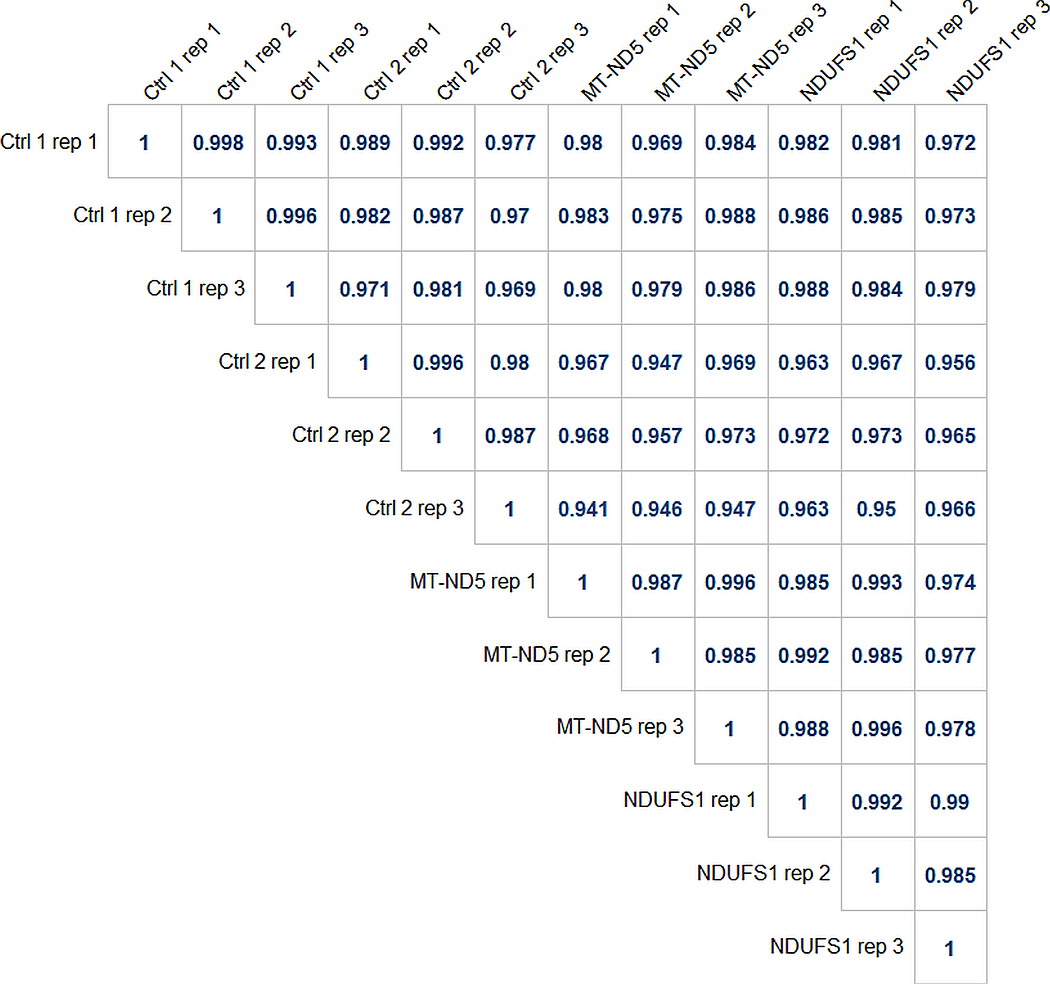

Supplement: Supplementary file 1 [file cells-08-01149-s001.zip › Supplemental Figure S3.tif]
